# A Review on the Application of Lignocellulosic Biomass Ash in Cement-Based Composites

**DOI:** 10.3390/ma16175997

**Published:** 2023-08-31

**Authors:** Liguang Sun, Congguang Yao, Aofei Guo, Zhenyun Yu

**Affiliations:** 1 School of Civil Engineering, Zhengzhou University, Zhengzhou 450001, China; 2 Zhengzhou Metro Group Co., Ltd., Zhengzhou 450001, China

**Keywords:** lignocellulosic biomass ash, cement-based composite, physicochemical property, mechanical property, durability

## Abstract

With the development of society, the demand for cement-based composites is increasing day by day. Cement production significantly increases CO_2_ emissions. These emissions are reduced when high volumes of cement are replaced. The consideration of sustainable development has prompted people to search for new cement substitutes. The lignocellulosic biomass ash obtained from burning lignocellulosic biomass contains a large number of active oxides. If lignocellulosic biomass ash is used as a partial cement substitute, it can effectively solve the high emissions problem of cement-based composites. This review summarizes the physicochemical properties of lignocellulosic biomass ashes and discusses their effects on the workability, mechanical properties, and durability (water absorption, acid resistance, etc.) of cement-based composites. It is found that appropriate treatments on lignocellulosic biomass ashes are beneficial to their application in cement-based composites. Meanwhile, the issues with their application are also pointed out.

## 1. Introduction

Cement concrete is well recognized as one of the major construction materials throughout the world. Cement production consumes a large amount of energy and emits carbon dioxide (CO_2_) into the atmosphere. It was reported that the production of cement accounts for over 4–7 percent of global CO_2_ emissions and poses severe environmental problems worldwide [1]. The production of 1 ton of cement released approximately 1 ton of CO_2_ [2]. The increase in CO_2_ emissions has led to the greenhouse effect and an increase in the Earth’s temperature. On 22 April 2016, the leaders from 175 countries signed the historic “The Paris Agreement” on Earth Day to slow the rise of greenhouse gases [3]. Moreover, during the production process of cement concrete, issues such as the consumption of aggregates and energy makes it a non-environmentally friendly material that is unsuitable for sustainable development. Hence, researchers have been studying the effectiveness, efficiency, and availability of supplementary cementitious materials (SCMs) as a cement replacement.

The use of supplementary cementitious materials (SCMs) to partially replace cement in concrete provides an alternative to reduce the use of cement. The most widely used SCMs include fly ash (FA), blast furnace slag (BFS), silica fume (SF), etc. These SCMs are pozzolanic materials. In the presence of moisture, the pozzolanic materials react with the calcium hydroxide (CH) which is produced from Portland cement hydration to produce a silicon- or aluminum-containing hydraulic compound with cementitious properties, which can strengthen cement-based composites [4,5]. SCMs are increasingly popular because they can not only effectively reduce the amount of cement used in the construction industry and thus reduce the overall environmental impact, but also improve various properties of concrete [6]. The partial replacement of cement with fly ash can improve the workability, long-term strength, and durability of concrete [7]. Moreover, concrete made with fly ash has a lower heat of hydration, which makes it ideal for mass concrete [8]. BFS [9] can reduce hydration heat and improve the pore structure of concrete. SF can fill the pores of the matrix and help form dense hydration products to improve the performance of concrete material [10]. Because the share of coal-based electricity generation is declining at a steady rate, the amount of fly ash is reduced; silica fume is too expensive to be used; and the alkali content in slag is high. Therefore, finding new supplementary cementitious materials is an alternative.

Biomass is an important renewable resource, which is sourced from agricultural waste, wood and forest waste, urban organic waste, algae biomass, etc. At present, the annual production of major biomass resources in China is about 3.494 billion tons, and the utilization of biomass resources is about 461 million tons, with a utilization rate of 13%, resulting in a CO_2_ reduction of about 218 million tons [11]. Among them, agricultural waste and wood and forest waste can be collectively referred to as lignocellulosic biomass. In some countries, lignocellulosic biomass is used as fuel for heat and power generation in industry. Lignocellulosic biomass ash usually refers to the ash produced by fully burning lignocellulosic biomass [12]. The production of these ashes may bring in some environmental issues. On one hand, when these ashes are released into the atmosphere, they deteriorate air quality and pose serious threats to human health by causing cardiovascular diseases [13]. On the other hand, these ashes are often disposed of in landfills or forest land, which not only causes waste of land resources but also affects groundwater safety [14,15,16]. Because lignocellulosic biomass absorbs a large amount of silicate from the soil during the growth process, there is some SiO_2_ in the chemical composition of lignocellulosic biomass ash, showing similar behavior to conventional supplementary cementitious materials such as fly ash and silica fume [17,18,19,20]. Thus, if lignocellulosic biomass ash can be used as a supplementary cementitious material in cement concrete, the issues caused by it can be effectively eliminated.

Research on lignocellulosic biomass ash as a mineral admixture began in the 1970s. Cook et al. [21] found that rice husk ash could constitute up to 60% of the total cementitious component in the mix to produce units that would satisfy the requirements for non-load-bearing masonry. James et al. [22] found that rice husk ash could improve the later strength and durability of concrete. So far, research on lignocellulosic biomass ash in building materials such as ordinary concrete, self-compacting concrete, permeable concrete, and high-performance concrete has made certain progress [23,24,25].

This review introduces various types of lignocellulosic biomass ash and its role in reducing CO_2_ emissions. The physical and chemical properties of these lignocellulosic biomass ashes are discussed. The influence of lignocellulosic biomass ashes on the mechanical properties and durability of cement mortar and concrete is discussed in detail. This review comprehensively discusses the impact of different types of lignocellulosic biomass ash on cement-based materials and analyzes the advantages of concrete containing lignocellulosic biomass ash compared to ordinary concrete in terms of environment, cost, and energy consumption. At the same time, this review emphasizes the challenges faced by the practical application of lignocellulosic biomass ashes in cement-based composites.

## 2. Agricultural Waste

Common agricultural waste includes rice husks, straws, banana leaves, sugarcane bagasse, palm kernels, and so on. Currently, a portion of lignocellulosic biomass is being used as fuel for power generation [26]. The combustion of lignocellulosic biomass produces two types of ash, biomass bottom ash (BBA) and biomass fly ash (BFA). BBA refers to the ash left at the bottom of the furnace after the combustion of lignocellulosic biomass. BFA refers to the ash on the upper part of the furnace after the combustion of lignocellulosic biomass [26]. Generally, the BFA is used as fertilizer due to its high content of Ca, P, K, and other substances, and the BBA is buried owing to no practical applications [27]. Most scholars confirmed that the content of active oxides (SiO_2_ + Fe_2_O_3_ + Al_2_O_3_) in these ashes is more than 70%, which is a good choice for pozzolanic materials [27]. Therefore, this review introduces the application of biomass bottom ash in cement mortar and concrete.

### 2.1. Rice Husk Ashes (RHA)

**Figure 1:**
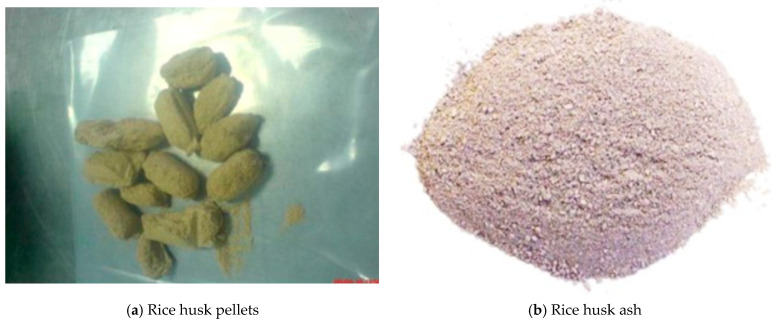
Appearance of (**a**) rice husk and (**b**) RHA [31,32].

In 2022, China’s rice production reached 212.13 million tons, producing over 42 million tons of rice husks [28]. Because rice husk is burned in open fields, various health and environmental problems usually appear. Rice husk ash (RHA) is the product of rice husk combustion. Research showed that the combustion of rice husks produced approximately 10–25% of ash [29,30]. Figure 1 shows the appearance of rice husk pellets and rice husk ash.

The chemical composition of RHA is investigated by researchers as shown in Table 1. The main chemical component of RHA is SiO_2_, with a content of over 80%. The alkali content in rice husk ash is high, which carries the risk of triggering the alkali–aggregate reaction. However, the K_2_O content can be reduced by controlling the combustion temperature and pre-treatment. Feng et al. [33] compared the oxide content of acid-treated RHA with that of untreated RHA and showed that the SiO_2_ content in acid-treated RHA increased by 3.60%, and the alkali content decreased. Bakar et al. [34] calcined RHA at 600 °C after acid treatment and found that the alkali almost disappeared. RHA can be classified as pozzolanic material because the percentage of SiO_2_ + Al_2_O_3_ + Fe_2_O_3_ is over 70% [35].

**Figure 2:**
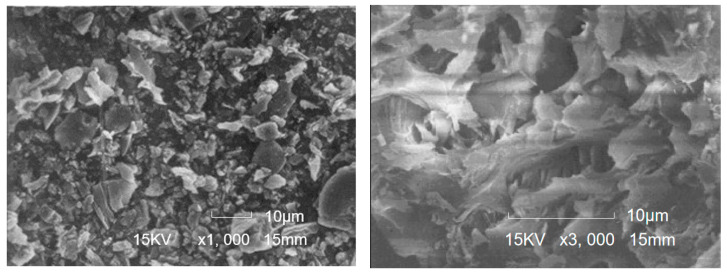
Microscopic appearance of RHA [47].

Figure 2 shows the microscopic appearance of RHA. RHA is a porous material. Therefore, RHA has a small particle size and a large specific surface area. Table 2 shows the physical properties of RHA. Ganesan et al. [43] reported that the median particle size, specific surface area, and specific gravity of RHA were 3.80 μm, 36.47 m^2^/g, and 2.06, respectively. Sinsiri et al. [38] and Muhammad et al. [39] found that the grinding treatment of RHA significantly affects its physical properties.

**Table 2:** Physical properties of RHA.

Ref.	Median Particle Size (μm)	Specific Gravity (g/cm^3^)	Specific Surface Area (m^2^/g)
[37]	9.17	2.17	31
[38]	14.8	2.29	-
	1.9	2.31	-
[39]	43.6	-	65.6
	17.3	-	55.6
	8.5	-	46.8
[42]	12.5	2.3	75.7
[43]	3.8	2.06	36.47
[44]	75–221	2.11	-
[45]	-	2.19	64.7
[46]	24.73	2.07	27.12

**Figure 3:**
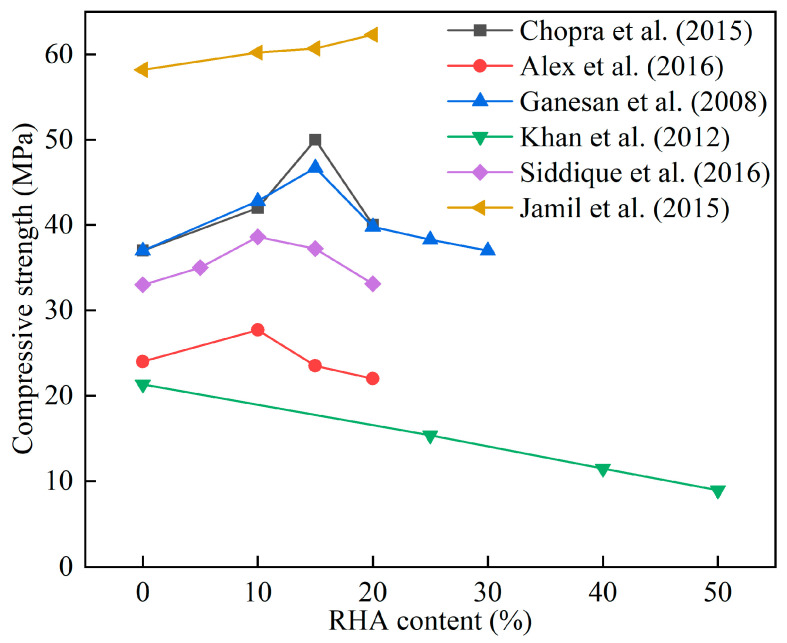
The effect of RHA content on the compressive strength of cement mortar and concrete [43,44,47,48,50,53].

Chopra et al. [48] found that due to the pozzolanic effect of RHA, 15% cement replacement could increase the compressive strength of concrete by more than 15% and improve the pore structure of concrete. Alex et al. [44] replaced the cement with 10% of RHA with different grinding degrees and found that the strength was improved to different degrees. The improvement in the strength was due to the high fineness of the RHA particles, which activated the pozzolanic property and improved the interfacial transition zone between aggregates and cement pastes. Huang et al. [45] found that replacing 2/3 silica fume with RHA to prepare ultra-high performance concrete increased the compressive strength by about 15% and 10% compared to the control group at 28 and 120 days, while the flexural strength was basically the same as the control group at 28 and 120 days. Sata et al. [49] made high-strength concrete using 10–30% RHA and found that the use of RHA to partially replace cement had no significant effect on splitting tensile strength as compared to control concrete. Siddique et al. [50] found that concrete containing 10% RHA exhibited higher compressive strength compared to ordinary concrete at different curing ages. Table 3 lists the effect of RHA on the compressive strength of cement mortar and concrete. Figure 3 shows the effect of RHA content on the 28 d compressive strength of cement mortar and concrete. It was found that the use of 10–20% RHA in most studies improved the compressive strength of cement mortar and concrete. Nguyen et al. [51] found that free water was absorbed into pores by RHA particles during hydration. As the cement hydration progressed, the relative humidity in the bulk cement slurry decreased, and the water in the RHA particles was released to enhance cement hydration [51].

**Table 3:** Effect of RHA on the mechanical properties of cement mortar and concrete.

Ref.	Replacement Level	Mix Type	Effect
[48]	0%, 10%, 15%, and 20%	self-compacting concrete (SCC)	Compared with the control group, the workability of SCC prepared by replacing some cement with 15% RHA decreased, and the compressive strength increased by about 36% at 56 days compared to the control group.
[45]	0, 1/6, 1/3, 1/2, 2/3, 5/6, and 1	ultra-high performance concrete (UHPC)	The use of RHA decreased the workability of UHPC mixtures and enhanced the compressive strength and impermeability.
[44]	10%, 15%, and 20%	concrete	The 10% RHA increased the compressive strength of concrete by 7.8% and the tensile strength by more than 50% at 14 d.
[43]	0–35%	cement mortar	The compressive strength of cement mortar prepared by replacing cement with 15% RHA reached 46.7 MPa at 28 d, compared to 37 MPa in the control group.
[47]	0%, 25%, 40%, and 50%	concrete	The strength reduction rate of concrete containing 25% RHA at 28 days did not exceed 2%.
[52]	0%, 20%, 35%, and 50%	recycled aggregate concrete (RAC)	The use of RHA reduced the early strength of RAC, and the 90 d compressive strength of RAC containing 20% RHA reached 87% of the control group.
[53]	2.5–20%	cement mortar	The 90 d compressive strength of cement mortar containing 2.5–20% RHA is higher than that of the control group.

Many researchers reported that the use of RHA had an impact on the durability of cement concrete. Nisar et al. [54] found the influence of RHA on the carbonation of concrete. The results indicated that as the RHA content varied from 0% to 20%, the carbonation depth of concrete gradually increased. The RHA was not conducive to the development of concrete carbonation resistance performance. However, Jittin et al. [55] reported that the addition of RHA resulted in a denser pore structure, which reduced the diffusion of CO_2_. Saraswathy et al. [56] tested the chloride ion permeability coefficient of concrete through a Rapid Chloride Permeability Test (RCPT) and found that replacement of RHA drastically reduced the coulomb values. When 25% RHA was incorporated, the coulomb decreased from 1161 C to 213 C, and the resistance to chloride ion corrosion of concrete was the best. Zareei et al. [57] used RHA with high SiO_2_ content (>85%) in the preparation of concrete, and found that the 25% RHA reduced the coulomb of concrete from 4306 C to 928 C, enhancing the resistance to chloride ion corrosion of concrete. Genesan et al. [43] used 20% RHA in concrete to reduce the water absorption of concrete from 3.76 to 2.20 at 90 d. Genesan et al. [43] also reported that the use of 30% RHA in concrete enhanced its resistance to chloride ion attack. Table 4 shows the effect of RHA content on the durability of cement mortar and concrete.

### 2.2. Palm Oil Fuel Ash (POFA)

**Figure 4:**
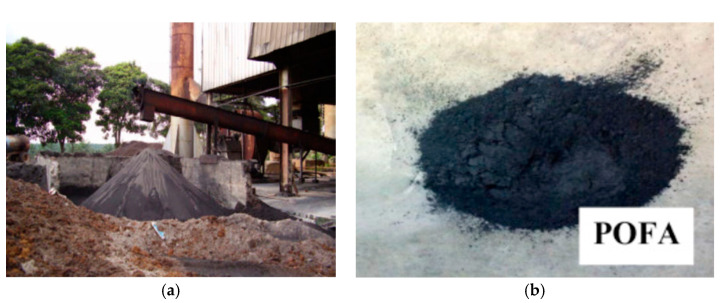
Production of POFA [62,63]. (**a**) Biomass factory. (**b**) Palm oil fuel ash.

As of 2022, the world’s palm oil production has reached 77 million tons and producing 1 ton of palm oil produces approximately 4 tons of palm oil biomass. POFA is a by-product material resulting from the burning of palm oil biomass (Figure 4). The palm oil biomass combustion generates approximately 10% of POFA [59]. POFA is usually disposed into open areas and landfill due to its lower nutrient value. Muthusamy et al. [60] reported that the lightweight concrete produced by replacing part of the cement with 20% POFA exhibited the highest compressive strength. Hamada et al. [61] reported that the 30% POFA in lightweight aggregate concrete improved the slump from 60 mm to 90 mm, and the compressive strength was improved compared to the control group.

The chemical composition of POFA was summarized as shown in Table 5. Significant differences in the chemical composition of POFA were observed in different studies. SiO_2_ in POFA varied from 43% to 65%. The remarkable differences are due to many factors such as temperature, growth environment, artificial conditions, and others [64,65].

**Figure 5:**
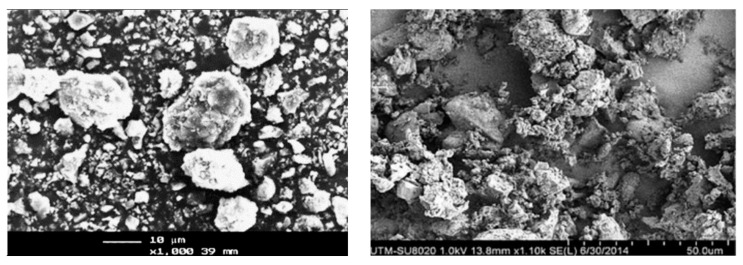
Microscopic appearance of POFA [49,75].

The physical properties of POFA are summarized in Table 6. Table 6 indicates that the specific gravity of POFA ranged between 1.8 and 2.6, and the specific surface area and the median particle size ranged within 1.4–15.0 m^2^/g and 1.07–23.0 μm, respectively. As shown in Figure 5, POFA was composed of irregular particles with angular and crushed shape through a scanning electron microscope (SEM) [49].

**Table 6:** Physical properties of POFA.

Ref.	Median Particle Size (μm)	Specific Gravity (g/cm^3^)	Specific Surface Area (m^2^/g)
[66]	13.0	2.22	-
[68]	-	2.42	-
[69]	15.6	2.36	6.7
	2.1	2.48	14.9
[71]	1.07	2.6	13.4
[72]	22.78	1.89	12.92
[73]	1.07	2.53	-
[74]	22.52	2.42	8.96
	22.53	2.56	5.56
[64]	2.45	2.5	1.694
	2.99	2.5	1.438
	2.06	2.6	1.775

Johari et al. [64] studied the effect of POFA on the setting time and workability of green concrete. The results indicated that when RHA varied 0% to 60%, the initial setting time of green concrete extended from 140 min to 350 min, and the final setting time increased from 285 min to 555 min, and the slump increased from 190 mm to 230 mm. Tangchirapat et al. [76,77] also found that the setting time increased with the increase of POFA in concrete.

**Figure 6:**
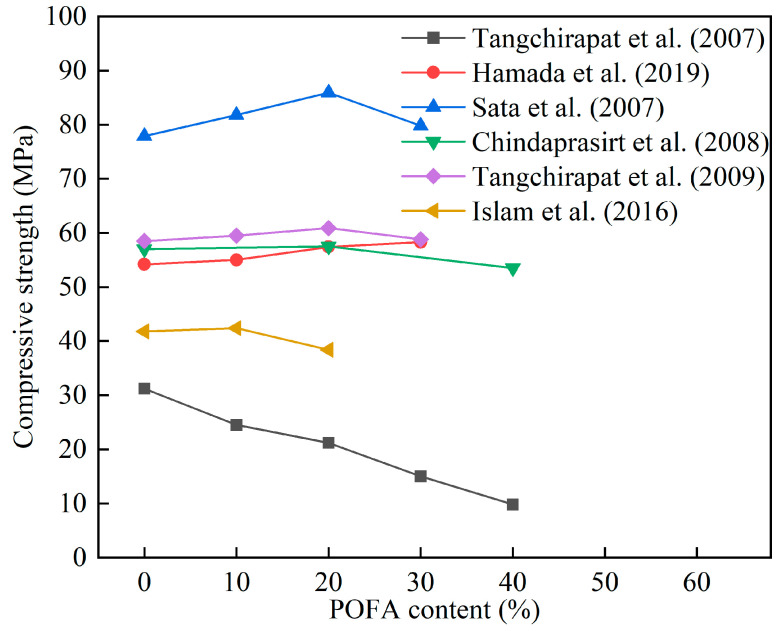
The effect of POFA content on the compressive strength of cement mortar and concrete [49,61,63,76,77,81].

Table 7 lists the effect of POFA on the compressive strength of cement mortar and concrete. Tangchirapat et al. [77] treated POFA to obtain three types of POFA with different median particle sizes, and found that the particle size and weight of POFA had an impact on the compressive strength of concrete. The compressive strength of concrete containing raw POFA was much lower than ordinary concrete. This was because the larger particle size of POFA was not conducive to the pozzolanic reaction. The 90 d compressive strength of concrete containing two other types of POFA were higher than ordinary concrete. Khankhaje et al. [78] applied POFA to pervious concrete and found that 10% POFA reduced the compressive strength and tensile strength of pervious concrete by 7% and 2%, respectively. Nevertheless, the strengths of POFA concrete were within the acceptable range of permeable concrete. Zeya et al. [79] produced high-strength concrete (HSC) using ultrafine POFA (UPOFA). It was found that HSC-UPOFA containing 20, 40, and 60% of UPOFA extended the compressive strength from 108.6 to 112.4 MPa, which was about 10% higher than that of high-strength concrete without POFA. Bellal et al. [80] calcined and ground POFA to reduce its median particle size, and used POFA as a cement substitute by 0, 30, 50, and 70% to study self-compacting concrete (SCC). It was confirmed that SCC containing 30% POFA significantly improved the compressive strength of SCC and exhibited similar fresh performance to the control SCC. Figure 6 shows the effect of POFA content on the 28 d compressive strength of cement mortar and concrete. Most studies confirmed that the use of 10–20% SCBA did not affect the compressive strength of cement mortar and concrete. Tangchirapat et al. [77] believed that untreated POFA had a larger particle size and could not promote the properties of concrete. The particle size of the treated POFA decreased, and its filling effect and pozzolanic properties enhanced the compressive strength of cement mortar and concrete.

The durability of cement concrete was improved due to the use of POFA. Tang et al. [82] investigated the influence of carbonation on concrete containing micro palm oil fuel ash (mPOFA) and nano palm oil fuel ash (nPOFA). The carbonation depth of concrete containing 10% mPOFA and 0.5% nPOFA at 70 d was 1 mm compared to 14 mm of ordinary concrete. Chindaprasirt et al. [81] showed that the addition of POFA, RHA, and FA improved the resistance of mortars to chloride penetration. RHA was the most effective, followed by POFA and FA. Alsubari et al. [83] found that the compressive strength loss rates of concrete containing 0%, 10%, 20%, 30%, and 50% POFA after acid attack were 18%, 14.7%, 13.0%, 12.5%, and 12.0%, respectively, and the use of 50% POFA reduced the quality loss of concrete from 2.8 to 1.84%. Ranjbar et al. [84] produced self-compacting concrete with 10%, 15%, and 20% POFA. It was found that the addition of POFA in self-compacting concrete reduced mass loss and strength loss under acid and sulfate erosion. Ranjbar et al. [84] believed this phenomenon was caused by the pozzolanic reaction between POFA and Ca(OH)_2_. Jaturapitakkul et al. [85] believed that POFA fineness was an important factor affecting the sulfate resistance of concrete. Jaturapitakkul et al. [85] replaced some cement with POFA of different finenesses and found that in a 5% magnesium sulfate solution, the addition of POFA reduced the expansion and strength loss of concrete. The durability of cement mortar and concrete containing POFA was shown in Table 8.

### 2.3. Corn Cob Ash (CCA)

**Figure 7:**
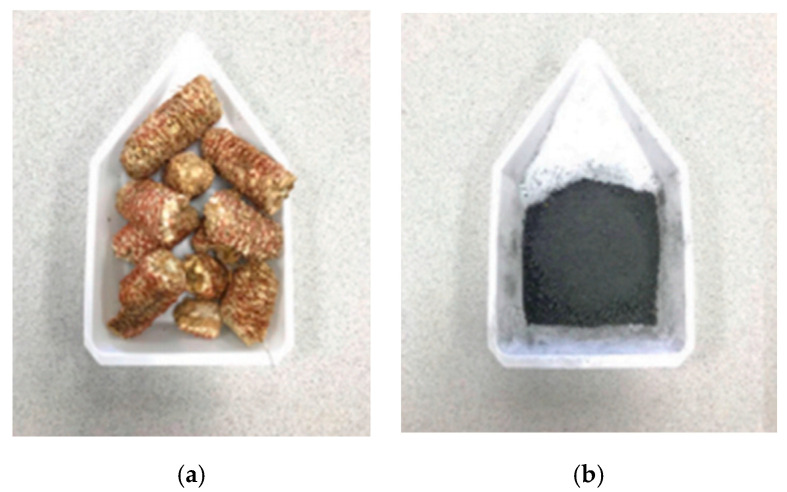
Raw corn cob and CCA [88]. (**a**) Corn cob. (**b**) Corn cob ash.

Corn is the most highly produced cereal, and its production exceeds wheat and rice. In 2022, China’s cereal production was 633.243 million tons, of which the total corn production was 277.23 million tons, accounting for approximately 44% of the total production [28]. In general, the corn cob is used as a biofuel [86]. The combustion of corn cob produced approximately 20–30% CCA [19]. As shown in Figure 7, corn cob ash (CCA) is gained from the combustion of corn cob which is an agricultural waste. Memon et al. [87] found that further incineration of CCA stimulated the pozzolanic property of CCA, which was a good choice to be used as a substitute for cement.

The chemical composition of CCA is listed in Table 9. It was found the active oxide (SiO_2_ + Al_2_O_3_ + Fe_2_O_3_) content is greater than 70%, which meets the ASTM C168 standard for cementitious material. Most of the CCA contains high alkali content (K_2_O), which causes the risk of an alkali–silica reaction. Prado et al. [37] reported that the potassium oxide content in CCA was reduced to 0.3% after acid leaching. Moreover, compared to natural CCA, leached CCA exhibited a higher specific surface area. The particle size, specific surface area, and density of CCA after acid treatment were 8.78 μm, 171.69 m^2^/g, and 2.08 g/cm^3^, respectively.

**Figure 8:**
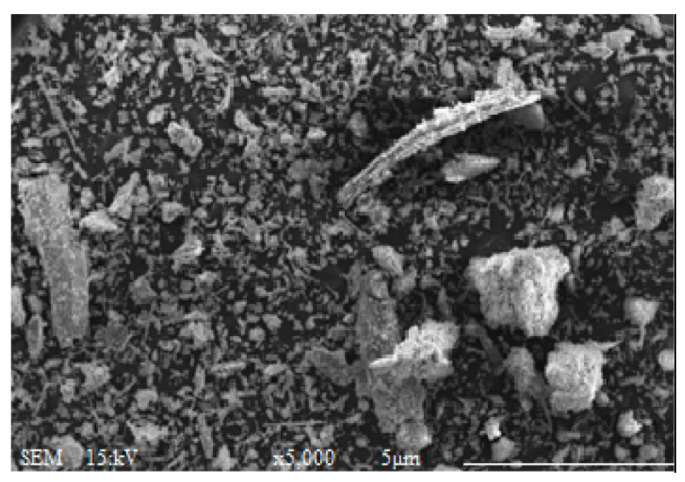
Microscopic appearance of CCA [94].

The physical properties of CCA are shown in Table 10. The specific gravity of CCA varied from 2 to 2.6. Memon et al. [19] investigated the physical properties of CCA with different grinding times and found the specific surface area of CCA increased with increasing grinding time. Prado et al. [37] subjected CCA to acid treatment and grinding, leading to a change in the specific surface area of CCA from 13.32 to 171.69. Figure 8 shows a microscopic appearance of CCA. The irregular particles were seen in Figure 8. The workability of concrete was reduced due to the sharp shape and rough surface texture of CCA. Adesanya and Raheem [89] indicated that with the increase of CCA, the slump of concrete decreased, which indicated that the workability of concrete was influenced by CCA.

The influence of CCA on the mechanical properties of cement mortar and concrete is shown in Table 11. The effect of using untreated CCA in the production of cement concrete is not good. Mahmoud et al. [88] found the untreated CCA was similar to an inert material through reactivity testing. Li et al. [90] used 2%, 4%, 6%, and 8% of CCA to replace part of cement, which resulted in reduced strength. This was due to the inertness and high alkali content of untreated CCA. The presence of basic oxide affected the cement hydration, resulting in low mechanical properties.

**Figure 9:**
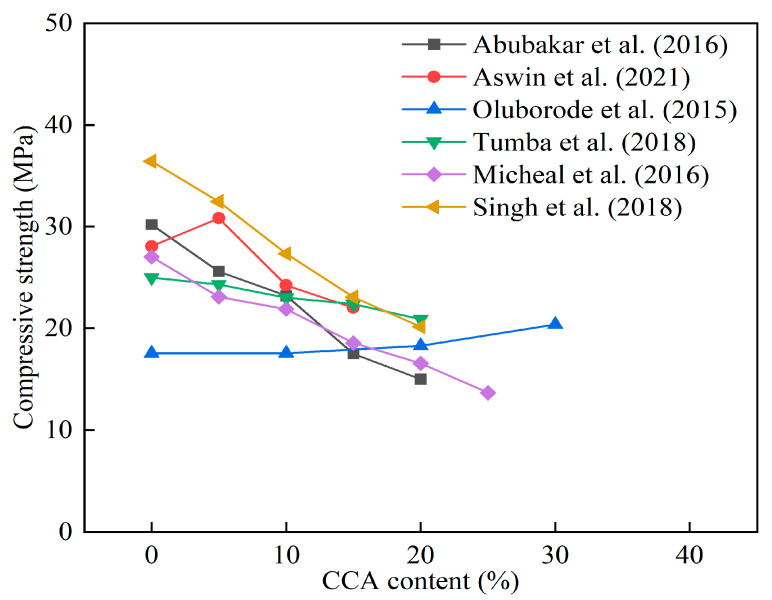
The effect of CCA content on the compressive strength of cement mortar and concrete [95,97,98,99,100,101].

Figure 9 shows the influence of CCA on the 28 d compressive strength of cement mortar and concrete. It was found that the use of CCA was not conducive to the development of compressive strength of cement mortar and concrete. Shakouri et al. [88] believed that the reason for this phenomenon was the low reactivity of untreated CCA. The CCA was not conducive to improving the compressive strength of concrete. However, the pretreatment CCA caused significant changes in the properties of cement-based composites. Pardo et al. [37] produced CCA by using acid leaching, calcination, and grinding. The mechanical properties of cement mortars with 20% and 30% treated CCA were higher than those of other cement mortars.

Table 12 shows the durability study of cement concrete with CCA partially replacing cement. Shakouri et al. [88] measured different ionic contents in the pore solution and found that the CCA in the concrete increased the concentration of free alkali metals, leading to an increase in chloride permeability. This was contrary to Adesanya et al.’s [102] finding that showed that the CCA reduced the water absorption capacity of concrete at lower CCA replacements, which resulted in reduced permeability. Adesanya et al. [102] indicated that a small amount of CCA played a filling role in the concrete to reduce the number of pores, while the excess CCA reduced the cement content. The filling effect of CCA could not compensate for the defects caused by the reduction of cement, so pores were generated in the mixture, leading to increased permeability. Kamau et al. [103] reported that the use of CCA enhanced the sulfate resistance of concrete. The application of CCA in insulation materials was also studied. Adesanya et al. [104] also found the addition of CCA in blended cement mortar improved the insulation properties of the material.

### 2.4. Sugarcane Bagasse Ash (SCBA)

**Figure 10:**
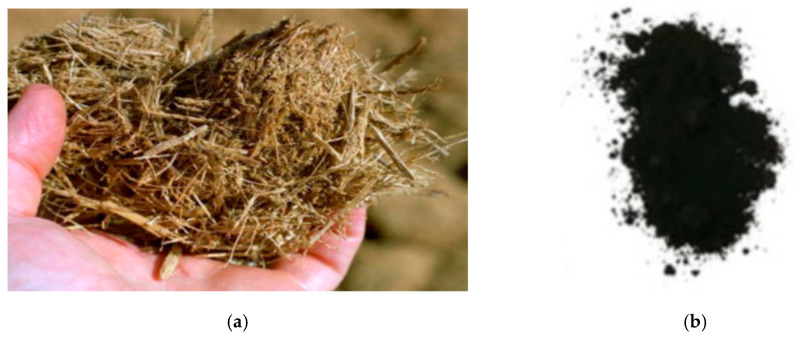
Appearance of sugarcane bagasse and SCBA [109,110]. (**a**) Sugarcane bagasse. (**b**) Sugarcane bagasse ash.

Sugarcane bagasse is from the sugar industry, obtained by extracting juice from sugarcane. According to research, 25% of the total mass of sugarcane produced is bagasse in the sugar industry. Bagasse is usually used as a biomass power plant, sugarcane bagasse contains more than 50% water, and after burning 1 ton of sugarcane bagasse, approximately 6.2 kg of SCBA is produced [107,108]. Therefore, sugarcane bagasse ash (SCBA) is obtained from power plants. Figure 10 shows the appearance of sugarcane bagasse and sugarcane bagasse ash.

The oxide contents of SCBA are listed in Table 13. The oxide contents of SCBA from different sources were different. SiO_2_ varied from 50% to 80%, as shown in Table 10. For a material to be considered as a pozzolan, the content of active oxides must be higher than 70%, according to ASTM C618 [35]. Moreover, the nature of silica determined the reactivity of SCBA. Some research reported that an amount of amorphous silica in SCBA imparted pozzolanic reactivity [111].

**Figure 11:**
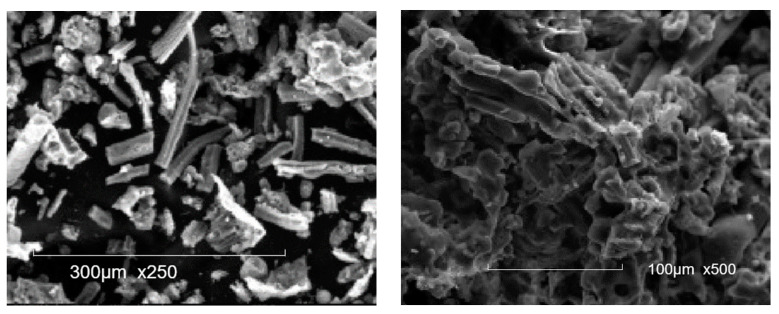
Microscopic appearance of SCBA [114].

Table 14 shows the physical properties of SCBA. The specific gravity of SCBA ranged between 1.9 and 2.3. The specific surface area of SCBA is less than 1 m^2^/g. Cordeiro et al. [111,116] reported that the specific surface area, density, and median particle size of SCBA were 196 m^2^/kg, 2530 kg/m^3^, and 76.3 μm, respectively. Cordeiro et al. [111] also analyzed that the amorphous material content in SCBA obtained at 700–900 °C was about 24%. Bahurudeen and Santhanam [114] reported that the burning of raw SCBA at 700–800 °C improved its pozzolanic activity. Hence, calcination could be used as one of the methods to stimulate the pozzolanic property of SCBA. The microscopic appearance of SCBA was shown in Figure 11. Irregular, prismatic, spherical, and fibrous particles were found in the microscopic appearance of SCBA [114].

**Figure 12:**
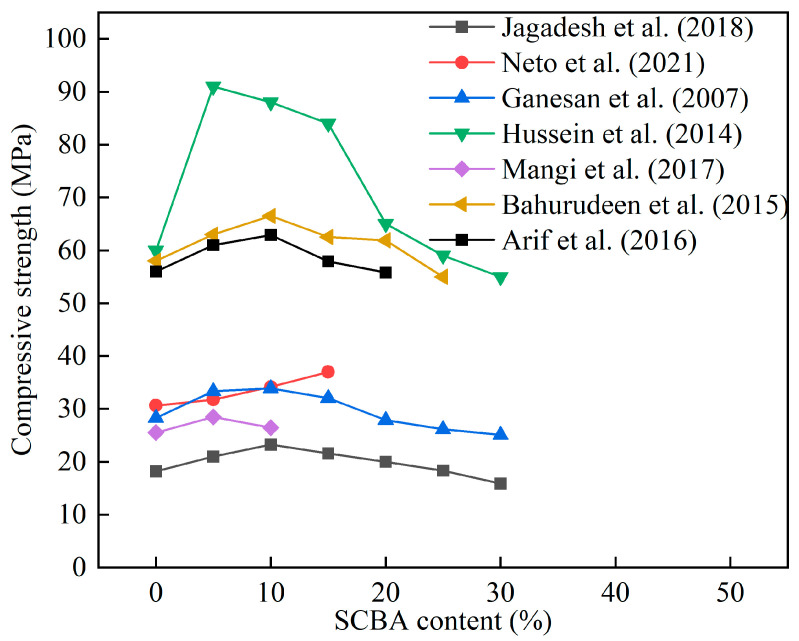
The effect of SCBA content on the compressive strength of cement mortar and concrete [113,119,122,123,124,128,129].

Figure 12 shows the influence of SCBA content on the 28 d compressive strength of cement mortar and concrete. The compressive strength of concrete was enhanced by 5–20% SCBA. Jagadesh et al. [119] found that untreated SCBA had a larger particle size, and its incorporation into cement concrete increased the porosity of concrete, resulting in decreased strength. The strength of SCBA was increased by 27% after pretreatment (grinding and burning), probably because grinding and calcination stimulated the activity of SCBA. Ganesan et al. [122] reported the influence of 5–30% SCBA content on the mechanical properties of concrete. The results showed that 20% SCBA increased the compressive strength and splitting tensile strength of concrete by 18% and 7% at 28 d, respectively. As the age increased, the pozzolan effect of SCBA improved the mechanical properties of the concrete [122]. Neto et al. [123] mixed 5%, 10%, and 15% SCBA into concrete and found that when the content was 15%, the SCBA reduced the porosity and improved the compressive strength of concrete. Hussein et al. [124] reported that the compressive strength of concrete containing 5–20% SCBA was higher than the control group. This change was attributed to the SCBA reducing the thickness of the interface transition zone between aggregate and paste. Moretti et al. [125] reported the application of SCBA in self-compacting concrete (SCC). The results confirmed that 30% SCBA did not have an impact on the workability and mechanical properties of SCC. Wu et al. [109] indicated that the addition of SCBA in UHPC not only improved the workability, but also decreased the autogenous shrinkage of UHPC paste. Table 15 shows the research on the influence of SCBA on the mechanical properties of cement mortar and concrete.

**Table 15:** Effect of SCBA on the mechanical properties of cement mortar and concrete.

Ref	Replacement Level	Mix Type	Effect
[119]	5–30%	concrete	Untreated SCBA reduced concrete strength, while 10% treated SCBA increased concrete strength by 27%.
[123]	5%, 10%, and 15%	concrete	15% SCBA in concrete led to a 20% increase in 28 d compressive strength compared to the control group.
[122]	5–30%	concrete	20% SCBA in concrete increased the compressive strength and splitting tensile strength by 18% and 7% at 28 d, respectively.
[125]	0%, 10%, and 20%	self-compacting concrete (SCC)	The changes in the workability and mechanical properties of SCC containing 30% SCBA were not obvious.
[109]	0–40%	ultra-high performance concrete (UHPC)	The compressive strength of UHPC containing 40% SCBA increased from 121.4 MPa to 130.8 MPa at 90 days, and the change in flexural strength was not obvious.
[126]	0% and 15%	cement mortar	The mechanical properties of concrete containing 15% SCBA from different sources were comparable to those of traditional concrete.
[127]	0%, 10%, and 20%	cement mortar	The SCBA reduced the workability of cement mortar and enhanced its compressive strength after 28 days.

Chopperla et al. [130] investigated the influence of SCBA on chloride resistance. The total charge passed was decreased by 74% with the addition of SCBA. According to Bahurudeen et al. [113], the total charge passed was reduced by 74% and 83% for the use of 15% and 25% SCBA compared to control group. Specimens containing SCBA showed higher chloride resistance than the control group. Murugesan et al. [131] used SCBA with a particle size less than 300 μm in concrete, and the use of 30% SCBA resulted in a 15% and 49% reduction in water absorption and water penetration depth, respectively. Rerkpiboon et al. [132] reported that replacing 50% cement with SCBA reduced the chloride penetration depth of concrete from 20 mm to 5 mm, and the expansion rate in sulfate decreased from 0.0361% to 0.0167%. The influence of SCBA on the mechanical property and durability of cement mortar was studied by Joshaghaniet et al. [133], and the results indicated that the SCBA improved the pore structure of cement mortar, reduced the proportion of harmful pores (>50 nm), and improved the chloride ion corrosion resistance of cement mortar. Table 16 shows the influence of SCBA on the durability of cement mortar and concrete.

### 2.5. Bamboo Leaf Ash (BLA)

**Figure 13:**
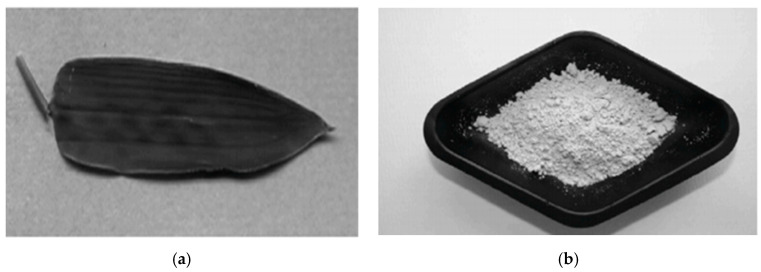
Appearance of a bamboo leaf and BLA [139]. (**a**) Bamboo leaf. (**b**) Bamboo leaf ash.

Bamboo is the highest production, and fastest growing building material. The production of bamboo is estimated to be 20 million t/year, and the total area of bamboo forests accounts for approximately 0.8% of the global land cover. However, 35–40% bamboo is usually incinerated in open-air landfills. The combustion of bamboo leaf can produce approximately 5–10% bamboo leaf ash [135,136,137]. Recent research [138] had shown that bamboo leaf ash (BLA) also had pozzolanic properties similar to RHA, which was one of the choices for SCMs. The appearance of bamboo leaves and bamboo leaf ash was shown in Figure 13.

Table 17 summarizes the chemical composition of BLA in recent years. X-ray fluorescence spectroscopy (XRF) analysis reveals that SiO_2_ was the major component (70–80%), followed by CaO (4–10%), K_2_O, and Al_2_O_3_. The loss of ignition (LOI) was above 4%. According to GB/T 51003, the ignition loss of pozzolanic material is less than 6%. Hence, Ernesto et al. [140] increased the calcination temperature from 500 °C to 700 °C, resulting in a decrease in the loss on ignition of BLA from 8.55% to 3.98%. Villar et al. [139] obtained BLA by calcining at 600 °C with a SiO_2_ content of up to 80%. Moraes et al. [141] measured the behavior of pozzolanic properties with the conductivity method and indicated that BLA was completely amorphous, exhibiting high pozzolanic activity.

**Figure 14:**
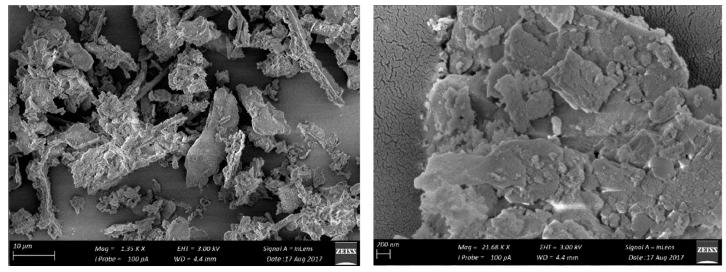
Microscopic appearance of BLA [143].

Kolawole et al. [143] analyzed the physical properties and microscopic appearance of BLA. The BLA had a similar fineness to Portland cement. The specific gravity and density of BLA were 2.00 and 0.6 g/cm^3^, respectively. X-ray diffraction (XRD) revealed that the main component of amorphous materials in BLA was SiO_2_. Figure 14 shows the microscopic appearance of BLA obtained at 1000 °C. BLA particles tend to be flat with more clusters [143].

**Figure 15:**
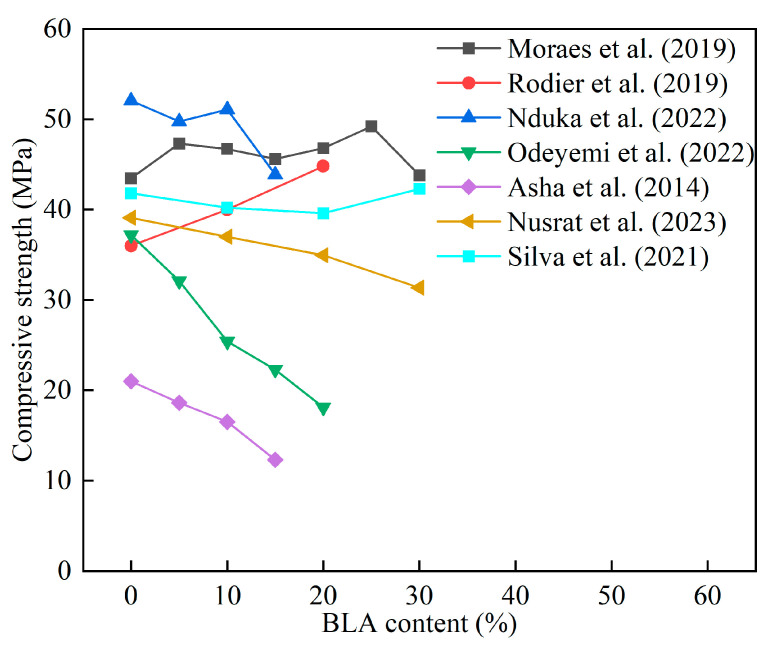
The effect of BLA content on the compressive strength of cement mortar and concrete [135,141,144,147,148,149,150].

Figure 15 shows the influence of BLA content on the 28 d compressive strength of cement mortar and concrete. It was found that the use of BLA did not lead to a decrease in concrete strength. Moraes et al. [141] found that the mechanical properties of cement mortar containing 5–25% SCBA were better than those of the control group. Rodier et al. [144] reported that BLA had high pozzolanic properties. The 7-day compressive strength of cement mortar containing 10% BLA was less than that of the control group, while the compressive strength increased by about 27% after 28 days. This indicates that replacing cement with BLA reduces the early strength, as the BLA diluted cement in the early stages [146]. Odeyemi et al. [147] reported that using 5% BLA in HPC increased the compressive strength by more than 30% at 56 days. Mim et al. [148] reported that the slump of self-compacting concrete (SCC) reduced as the BLA content increased, and the mechanical properties of BLA mixed SCC slightly decreased. Nduka et al. [149] obtained BLA by calcining elephant grass at 600 °C for 2 h, and showed the increase of BLA decreased the early compressive strength of the specimens. High-performance concrete (HPC) with 10% BLA had a similar compressive strength to that of the control group at 60 d. Table 18 summarizes the research on BLA in cement mortar and concrete.

There is limited research on the durability of BLA in cement-based material. Table 19 summarizes the impact of BLA on the durability of cement mortar and concrete in recent years. Dacuan et al. [152] evaluated the influence of BLA on the acid resistance of concrete. The results indicated the BLA had adverse effects on the compressive strength of concrete. However, the use of 10% BLA resulted in almost no loss in the quality and strength of the concrete after acid attack. The compressive strength of concrete with 20% BLA replacement decreased by 14.22% after acid erosion. Temitope et al. [153] reported the sulfate resistance of concrete mixed with BLA and reported that 10% BLA was suitable. Nehring et al. [154] indicated that the mass losses of cement mortar replaced by 20% BLA and 30% BLA after acid erosion were less than those of the control group. However, compared to cement mortar, the mechanical properties of cement mortar with 20% BLA and 30% BLA after acid erosion decreased.

### 2.6. Elephant Grass Ash (EGA)

**Figure 16:**
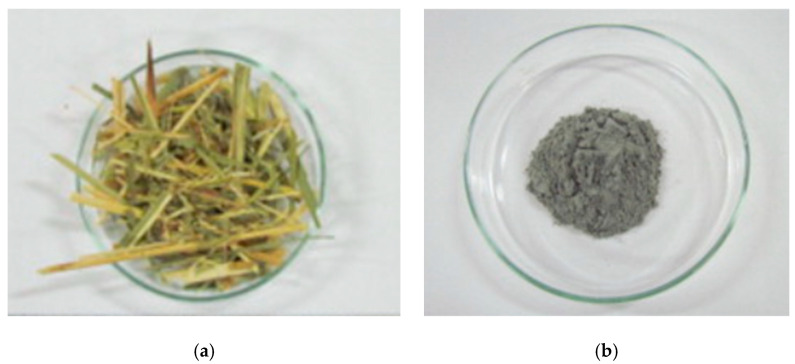
Appearance of elephant grass and EGA [157]. (**a**) Elephant grass. (**b**) Elephant grass ash.

Elephant grass, also known as *Pennisetum purpureum*, is usually used as animal feed [155]. In addition, Brazil produces a large amount of charcoal and bio-oil from elephant grass due to the large production of elephant grass [155]. Currently, Brazil uses 100% of elephant grass as a fuel source in power plants, which produces a large amount of ash during the burning process leading to some environmental problems. The production of elephant grass ash (EGA) is about 4–5% of the total grass mass. Because elephant grass absorbs silicon from the soil, it contains a considerable amount of amorphous silicon dioxide, which gives it pozzolanic properties similar to RHA and SCBA [156]. Figure 16 shows the appearance of elephant grass and elephant grass ash.

Table 20 shows the main chemical components of EGA. The oxidation composition of elephant grass ashes is mainly SiO_2_ (>50%), K_2_O (3–10%), and CaO (<10%). The Na_2_O content is very low. Cordeiro et al. [158] found that the SiO_2_ content in EGA gradually increased with the increase of calcination temperature, and the loss on ignition gradually decreased, reaching the optimal value at 800 °C. The SiO_2_ content and ignition loss were 56.4% and 1.6%, respectively. Nakanishi et al. [159] increased the calcination temperature of EGA from 700 °C to 900 °C and found that the silica content increased from 50% to 70%. Frias et al. [142] confirmed through XRD that there were a large number of amorphous phases in EGA, which gave EGA pozzolanic properties.

**Figure 17:**
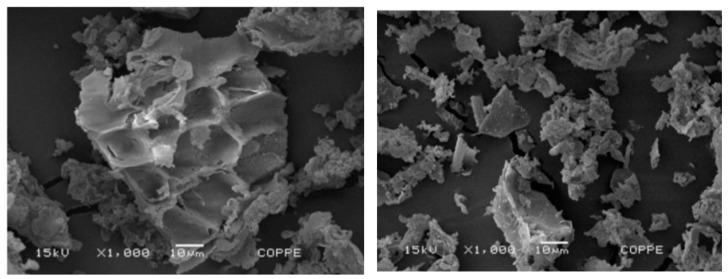
Microscopic appearance of EGA [156].

Table 21 shows the physical properties of EGA. Cordeiro et al. [156] investigated the physical properties and microscopic appearance of EGA. The specific gravity and median particle size of EGA were 2.51–2.62 and 10–11.6 μm, respectively. The specific surface area of EGA was 42–44 m^2^/g. The specific surface area of EGA after acid treatment reached 72.6 m^2^/g. The increase of the specific surface area meant that the surface of the pozzolanic reaction was larger, significantly increasing reactivity. Cordeiro et al. [158] also found that calcination of EGA can increase the median particle size of EGA and reduce its specific surface area. From Figure 17, it was found the EGA obtained by combustion at 600 ° C has different shapes, including porous and flaky particles [156].

The pozzolanic property of EGA enables it to be well applied in cement mortar and concrete. Table 22 presents the influence of EGA on the mechanical properties of cement mortar and concrete. Nakanishi et al. [160] obtained EGA by calcining elephant grass at 600 °C for 2 h. The study indicated that the compressive strength of cement containing 20% EGA decreased by 17% compared to the control group after 90 days of curing period. Canseco et al. [162] reported that EGA was suitable for concrete hollow blocks (CHB). The slump values of 0%, 10%, 15%, and 20% EGA substitutes were 12 mm, 10 mm, 9 mm, and 5 mm, respectively. The compressive strength of CHB containing 20% EGA was 3.91 MPa, which was significantly improved compared to the control group’s 2.7 MPa.

The research about the durability of cement concrete with EGA was limited. Most durability issues are caused by ion erosion, and the main transport medium for harmful ions to invade concrete structures is water. Some studies [156,164] showed that EGA had almost no effect on the water absorption of concrete. Hence, EGA may have minimal impact on the durability of cement and concrete.

### 2.7. Other Lignocellulosic Biomass Ashes

In addition to the aforementioned lignocellulosic biomass ashes, research has also been done on the application of corn stalk ash (CSA), rice stalk ash (RSA), wheat straw ash (WSA), and others in concrete.

**Figure 18:**
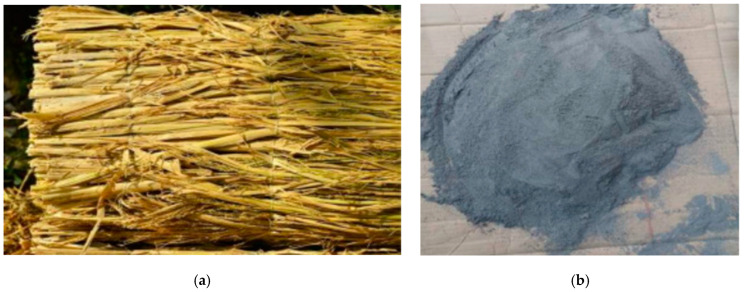
Dried corn stalk and CSA [166,167]. (**a**) Corn stalk. (**b**) Corn stalk ash.

**Figure 19:**
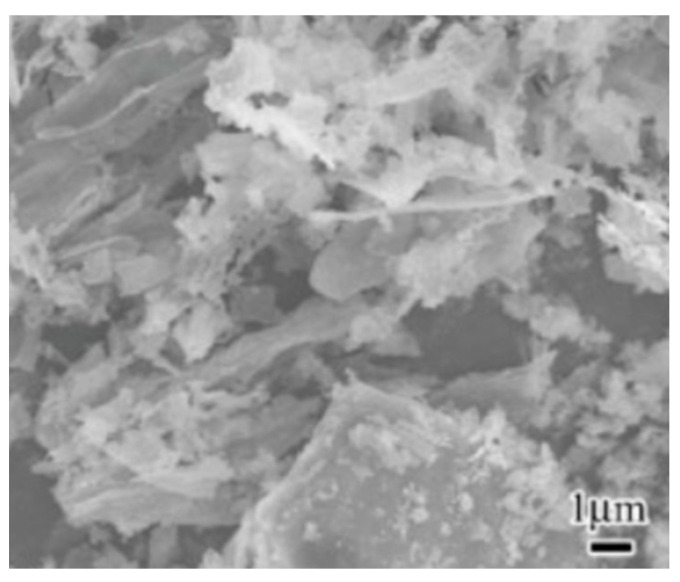
Microscopic appearance of CSA [90].

Corn straw is an agricultural byproduct (Figure 18). Rahemm et al. [165] calcined corn stalks to obtain corn stalk ash (CSA). The total SiO_2_, Fe_2_O_3_, and Al_2_O_3_ in CSA was over 70%. Figure 19 shows the microscopic appearance of CSA, and it can be seen that CSA has various shapes such as fibrous particles and flaky particles. CSA and cement were used to produce interlocking paving stones. Rahemm et al. reported that 5–25% CSA reduced the early strength of interlocking paving stones; the strength of interlocking paving stones prepared with 10% CSA was higher than that of the control group [165].

**Figure 20:**
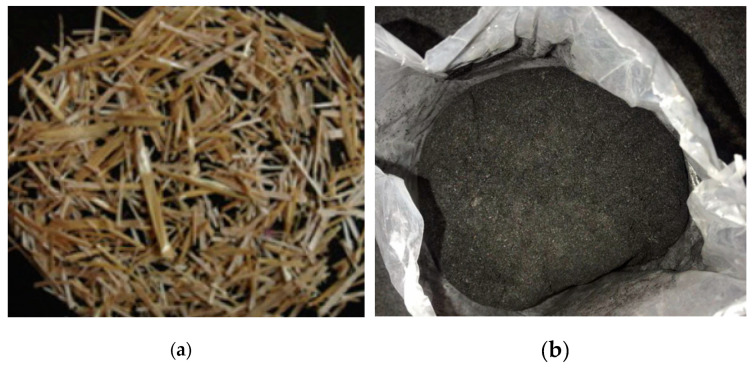
The appearance of wheat stalk and WSA [170,171]. (**a**) Wheat stalk. (**b**) WSA.

**Figure 21:**
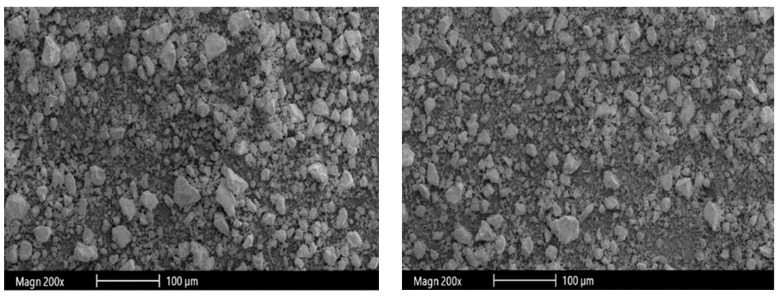
Microscopic appearance of WSA [168].

The production of wheat in China was approximately 137.72 million tons in 2022 [28]. Wheat straw is agricultural waste obtained from wheat seeds obtained from grains. Wheat straw ash (WSA) with pozzolanic properties was gained by burning wheat straws. Figure 20 shows the appearance of wheat stalk and WSA. Qudoos et al. [168] calcined wheat straws at 670 °C for 5 h to obtain wheat straw ash (WSA). The active oxide content (SiO_2_ + Fe_2_O_3_ + Al_2_O_3_) in WSA reached over 70%. As shown in Figure 21, various prismatic, spherical, and fibrous particles were shown in WSA. The compressive strength of cement mortar containing 20% WSA reached 80.7 MPa after 90 days, which was about 13% higher than the 71.2 MPa of the control group, 30% WSA significantly decreased the strength of cement mortar [168]. Biricik et al. [169] reported that the compressive strength of concrete containing 8% WSA at 28 days reached 89% of that of the control group, and the difference in compressive strength gradually decreased as curing age increased.

**Figure 22:**
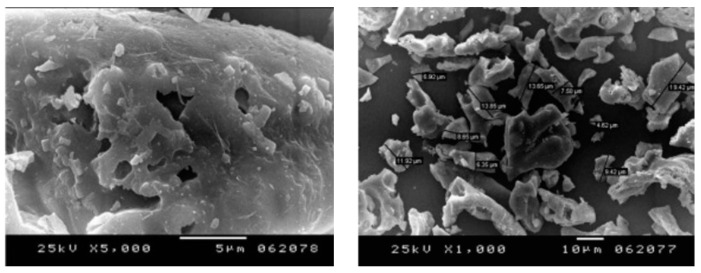
Microscopic appearance of RSA [174].

Mohamed et al. [172] reported that every 1000 kg of rice straws produces about 150 kg RSA after burning, with 82% SiO_2_, a specific surface area of 18,460 cm^2^/g, and a specific gravity of 2.25. The microstructure of RSA was microporous and irregular, as shown in Figure 22. Amin et al. [173] used 0–50% RSA to prepare ultrahigh-performance concrete (UHPC) and the slump values of the samples were 375 mm, 365 mm, 360 mm, 353 mm, 344 mm, and 336 mm, respectively, confirming that the use of RSA reduced the workability of UHPC. The compressive strength of UHPC containing 20% RSA reached 217 MPa at 91 d, which was about 14% higher than the 191 Mpa of the control group. RSA contents of 5%, 10%, and 20% were used in the preparation of lightweight self-compacting concrete (LWSCC) by Awga et al. [174]. The use of 10% RSA increased the compressive strength of LWSCC from 29 Mpa to 31 Mpa and the flexural strength from 5.4 Mpa to 6.6 Mpa. Awga et al. [174] believe that the main reason of the change in strength is the filler effect of RSA.

Wood ash (WA) is mainly obtained from wood or charcoal burned in rural cooking. The total content of SiO_2_, Al_2_O_3_, and Fe_2_O_3_ in wood ash is 73% through XRF detection, which is a good choice for pozzolanic materials [175]. WA content of 5–25% was used in concrete by Rahemm et al. [175]. The slump of concrete first decreased and then increased with the increase of WA. The 5% and 10% wood ash replacements resulted in compressive strength values of 19.10 MPa and 21.11 MPa at 28 d, respectively, which were greater than those of the control concrete (18.44 MPa). The compressive strength of the control group was better than that of the other groups at 120 d. These changes were attributed to the reaction of WA with Ca(OH)_2_ released during cement hydration. This was consistent with previous studies on pozzolanic ash [89].

Corn husk is the enclosure wrapped around corn, which is a type of agricultural waste. Corn husk ash (CHA) was obtained by burning corn husks. The percentage of active oxides of CHA obtained by Ndububa et al. [176] through calcination and grinding is 80%. The use of 5% CHA increased the compressive strength of concrete from 11.45 MPa to 14.96 MPa at 7 d [176].

### 2.8. Discussion

Different lignocellulosic biomass ashes have different effects on cement mortar and concrete. RHA significantly enhances the mechanical properties and durability of cement mortar and concrete, but the workability of concrete is reduced due to the high SiO_2_ content and high specific surface area of RHA. CCA tends to be an inert material. CCA has a negative impact on the properties of concrete due to its high alkaline oxide content. It is usually activated by acid pickling and calcination to reduce its negative effects on concrete. The production of SCBA is much lower than that of other lignocellulosic biomass ashes due to the higher water content in sugarcane bagasse, and SCBA has a smaller specific surface area compared to other lignocellulosic biomass ashes. The influence of SCBA on the workability of concrete can be ignored. The production of BLA and POFA is about 10% of lignocellulosic biomass. BLA and POFA have little influence on the properties of concrete. There is relatively little research on EGA, WSA, RSA, CSA, WA, and CHA. From existing research, it has been found that the use of a small amount of these biomass ashes does not have an obvious influence on the mechanical properties of cement mortar and concrete.

Some research examined the effects of lignocellulosic biomass ash on energy conservation, emission reduction, and production cost reduction. In terms of energy consumption, according to literature [177], during the production of cement, a high temperature of around 1450 °C is required, while the production of lignocellulosic biomass ash only needs 600–800 °C. In terms of cost-effectiveness, the price of cement is approximately 90 US dollars per ton, while only transportation costs need to be considered for lignocellulosic biomass ash. In terms of environmental impact, research showed that producing 1 ton of cement released 1 ton of CO_2_, but the production of lignocellulosic biomass ash hardly released CO_2_, as the released CO_2_ could be absorbed throughout the plant’s lifecycle [144]. Siliva et al. [135] evaluated the cost-effectiveness and economic benefits of producing ordinary concrete and concrete containing 30% lignocellulosic biomass ash based on the lifecycle of lignocellulosic biomass ash. Research showed that producing 1 m^3^ of ordinary concrete consumed approximately 2884 MJ energy and released 533 kg CO_2_. However, producing 1 m^3^ of concrete containing 30% lignocellulosic biomass ash consumed 2069 MJ energy, which is a reduction of about 28% compared to ordinary concrete, and released 375 kg CO_2_, which is a reduction of about 29% compared to ordinary concrete. The production cost also decreased from 67.98 US dollars to 57.86 US dollars. Rodier et al. [144] also evaluated the potential benefits of using lignocellulosic biomass ash in cement concrete. The results showed that the use of 20% lignocellulosic biomass ash in concrete reduced the production energy consumption from 112 kWh/t to 98.56 kWh/t. Overall, the application of lignocellulosic biomass ash in concrete can reduce energy consumption, CO_2_ emissions, and production costs of concrete.

## 3. Challenges for Future Application

With the progress of society, sustainable development has become the theme of the world. The high CO_2_ emissions caused by cement production have become a problem that cannot be ignored. The effective utilization of lignocellulosic biomass ashes is a good choice to solve this problem. However, there are still the following problems for lignocellulosic biomass ashes to become a marketable mineral admixture such as FA and BFS.

Firstly, the utilization rate of lignocellulosic biomass is only 13% in China [11]. Lignocellulosic biomass ash comes from lignocellulosic biomass combustion, and the mass production of lignocellulosic biomass ashes consumes a large amount of energy. Therefore, if the application of lignocellulosic biomass in power plants can be popularized, the above problems can be effectively solved.

Secondly, existing research has shown that due to the high specific surface area of lignocellulosic biomass ash, the use of lignocellulosic biomass ash can reduce the workability of concrete, which is not conducive to its practical application in engineering [24,27]. Therefore, the compatibility between superplasticizer and lignocellulosic biomass ash needs to be considered.

Thirdly, the impact of lignocellulosic biomass ashes on cement-based composites is influenced by the type of lignocellulosic biomass. For example, RHA enhanced the mechanical properties and durability of concrete; in contrast, CCA resulted in a decrease in the mechanical properties of concrete. Therefore, it may be necessary to distinguish the applications of various types of lignocellulosic biomasses in different situations.

Finally, lignocellulose biomass ash is a waste generated from the use of lignocellulosic biomass as fuel. Its main chemical components are oxides, including SiO_2_, CaO, and Al_2_O_3_. Therefore, lignocellulosic biomass ash is not hazardous. However, there is no literature on how to treat concrete containing lignocellulosic biomass ash at the end of its lifespan. After the end of the lifespan of concrete containing lignocellulosic biomass ash, using it as recycled aggregate may be a good solution.

## 4. Conclusions

This review focuses on the research on lignocellulosic biomass ash in cement mortar and concrete. The existing studies confirm the feasibility of using lignocellulosic biomass ash, such as RHA, CCA, BLA, SCBA, etc., as partial cement substitutes in cement mortar and concrete.

The content of SiO_2_ in RHA is above 80%. The early strength of cement mortar and concrete prepared by replacing 5–20% cement with RHA may be decreased. However, the use of RHA can enhance the later strength of cement mortar and concrete due to its pozzolanic nature. RHA has a positive influence on the durability of cement mortar and concrete.

The content of SiO_2_ in POFA is between 40 and 70%. The optimal content of POFA to replace cement is 10–20%. The use of POFA does not have an obvious influence on the mechanical properties but can significantly enhance the durability of cement mortar and concrete.

The application of CCA in cement mortar and concrete is terrible because CCA reduces the mechanical properties and durability of cement mortar and concrete. Although CCA has a silicon dioxide content of over 60%, untreated CCA has a higher alkaline oxide content. Acid-treated and calcinated CCA can replace 10–30% of cement without affecting the concrete performance.

The content of SiO_2_ in SCBA is over 50%, and its specific surface area is much smaller than those of other lignocellulosic biomass ashes. SCBA at 5–25% can enhance the mechanical properties and durability of cement mortar and concrete. Unlike RHA, the use of SCBA does not affect the workability of concrete.

The SiO_2_ content in EGA, WSA, RSA, CSA, WA, and CHA is above 50%. The use of these lignocellulosic biomass ashes in cement mortar and concrete does not cause a significant decrease in mechanical properties and durability. The workability of concrete was reduced by these lignocellulosic biomass ashes.

At the same time, the application of lignocellulosic biomass ash in cement mortar and concrete was analyzed from the aspects of environment, cost, and energy consumption, confirming that the application of lignocellulosic biomass ash in concrete can reduce the impact of concrete production on the environment and promote sustainable development.

## Figures and Tables

**Table 1:** Chemical composition of RHA.

Ref.	SiO_2_	Al_2_O_3_	Fe_2_O_3_	CaO	MgO	Na_2_O	K_2_O	SO_3_	LOI (%)
[36]	95.41	-	0.82	0.00	1.24	0.22	1.65	0.07	0.77
[37]	91.40	0.10	-	1.30	-	-	1.80	1.90	3.30
[38]	87.80	0.90	0.50	1.20	0.60	0.2	2.20	0.10	5.20
[39]	80.20	3.20	2.39	1.51	0.59	0.34	1.80	-	9.30
[40]	94.60	0.10	-	0.80	-	0.10	1.30	0.40	1.30
[41]	82.14	1.34	1.27	1.21	1.96	0.14	2.09	0.17	-
[42]	95.10	0.30	0.20	1.50	0.60	0.06	2.30	-	0.80

**Table 4:** Effect of RHA on the durability of cement mortar and concrete.

Ref.	Replacement Level	Effect
[56]	0–30%	When 25% RHA was incorporated, the coulomb decreased from 1161 C to 213 C.
[54]	0%, 5%, 10%, 15%, and 20%	With the increasing of RHA content, the carbonation depth of concrete increased.
[43]	0–35%	Replacing cement in concrete with 20–30% RHA did not have adverse effects.
[58]	5%, 10%, 15%, 20%, 25%, and 30% (by volume)	Replacing cement in cement mortar with 20% RHA reduced water absorption by 14% and the nonsteady state migration coefficient by 60%.
[57]	0%, 5%, 10%, 15%, 20%, and 25%	The 25% RHA produced 78% reduction in chloride permeation of concrete compared to ordinary concrete.

**Table 5:** Chemical composition of POFA.

Ref.	SiO_2_	Al_2_O3	Fe_2_O_3_	MgO	CaO	Na_2_O	SO_3_	K_2_O	LOI (%)
[38]	55.70	0.90	2.00	5.10	12.50	-	2.90	11.90	4.70
[66]	65.30	2.56	1.98	3.08	6.42	-	0.47	5.72	10.05
[67]	57.80	2.30	9.60	1.40	3.60	0.56	-	3.50	20.70
[60]	51.55	4.64	8.64	2.44	5.91	-	0.61	5.50	5.00
[68]	43.60	8.50	10.10	5.80	8.40	-	2.80	3.50	18.00
[69]	55.70	0.90	2.00	5.10	4.10	9.15	1.59	11.11	1.30
[70]	51.26	2.75	2.13	5.78	6.53	0.15	3.34	13.10	9.73

**Table 7:** Effect of POFA on the mechanical properties of cement mortar and concrete.

Ref.	Replacement Level	Mix Type	Effect
[77]	10%, 20%, 30%, and 40%	concrete	Raw POFA was not suitable in concrete.
[79]	20%, 40%, and 60%	high-strength concrete (HSC)	HSC with 40% treated POFA achieved 112.4 MPa at 180 d, which was higher than the control group (105.1 MPa).
[61]	0%, 10%, 20%, and 30%	lightweight aggregate concrete	The compressive strength of concrete containing 30% POFA was 58.3 MPa.
[80]	0%, 30%, 50%, and 70%	self-compacting concrete	The fresh properties of concrete with 30% POFA were similar to those of the control sample.
[49]	0%, 10%, 20%, and 30%	high-strength concrete	The compressive strength of concrete containing 10–30% POFA was higher than ordinary concrete at 7 d.
[81]	0%, 20%, and 40%	cement mortar	Replacing 20% cement with POFA had no impact on the strength of cement mortars.
[63]	0%, 5%, 10%, 15%, 20%, and 25%	lightweight concrete	At 5–15%, POFA had no significant impact on the lightweight concrete.

**Table 8:** Effect of POFA on the durability of cement mortar and concrete.

Ref.	Replacement Level	Effect
[82]	10%, 20%, and 30% mPOFA; 0.5%, 1.0%, and 1.5% nPOFA	The use of 1% nPOFA and 10% mPOFA improved the carbonation resistance the most among all concrete mixtures.
[83]	0%, 10%, 20%, 30%, and 50%	The use of 50% POFA in concrete reduced the compressive strength loss to 12% after acid attack, compared to 18% of ordinary concrete.
[67]	0%, 5%, 10%, 15%, and 20%	The samples containing POFA had better carbonization resistance than the control samples.
[81]	0%, 20%, and 40%	The samples with POFA had good strength and resistance to chloride penetration.
[84]	10%, 15%, and 20%	The use of POFA enhanced the durability of self-compacting concrete without affecting its strength.
[80]	0%, 30%, 50%, and 70%	The addition of 30% POFA reduced the chloride ion penetration at all levels.
[85]	0%, 10%, 20%, 30%, and 40%	The treated POFA can be used as a pozzolanic material, improving the sulfate resistance of concrete and reducing the loss rate of concrete strength.

**Table 9:** The chemical composition of CCA.

Ref.	SiO_2_	Al_2_O3	Fe_2_O_3_	CaO	MgO	Na_2_O	K_2_O	SO_3_	LOI (%)
[37]	62.50	-	0.90	8.70	-	-	17.20	2.60	4.00
[37]	94.20	-	0.20	0.40	-	-	0.30	1.60	1.9
[89]	66.38	7.48	4.44	11.57	2.06	0.41	4.92	1.07	-
[87]	63.73	15.05	5.32	6.56	4.56	0.10	2.05	-	2.30
[90]	64.80	2.90	9.40	5.00	5.10	-	6.50	0.70	1.50
[91]	62.30	6.25	4.40	10.57	1.86	0.36	3.89	-	-
[92]	64.90	10.79	4.75	10.24	2.08	0.43	4.23	2.53	-
[93]	62.34	9.55	10.16	12.65	1.33	0.55	1.15	1.30	2.95

**Table 10:** Physical properties of CCA.

Ref.	Median Particle Size (μm)	Specific Gravity (g/cm^3^)	Specific Surface Area (m^2^/g)
[94]	28.23	2.18	0.653
[37]	8.78	2.08	171.69
	9.23	2.36	13.32
[95]	-	2.27	-
[96]	-	2.54	-
[19]	10–54	-	1.321
		-	2.98
		-	4.22
		-	4.56

**Table 11:** Effect of CCA on the mechanical properties of cement mortar and concrete.

Ref.	Replacement Level	Mix Type	Effect
[88]	3% and 20%	concrete	Untreated CCA had low reactivity and was not suitable in concrete.
[90]	2%, 4%, 6%, and 8%	fly ash/cement composite	The use of untreated CCA reduced mechanical properties.
[97]	0%, 5%, 10%, 15%, and 20%	concrete	The compressive strength decreased with the increase of CCA content.
[37]	0%, 10%, 20%, and 30%	cement mortar	The mechanical properties of cement mortar with 20% and 30% treated CCA were higher than those of other cement mortars.
[98]	0–17.5%	ordinary concrete	The slump of concrete decreased with the increase of CCA, and 5% CCA content increased the strength of concrete by 10% compared to ordinary concrete.
[95]	0%, 10%, 20%, and 30%	self-compacting concrete (SCC)	The compressive strength of SCC with 30% CCA was 20.37 MPa, and the flowability and filling properties were 755 mm and 29 mm, meeting the requirements of SCC.

**Table 12:** Effect of CCA on the durability of cement mortar and concrete.

Ref.	Replacement Level	Effect
[88]	3% and 20%	The resistance of untreated CCA concrete to various ion erosion was lower than that of ordinary concrete.
[102]	0–25%	Lower levels (<15%) of CCA replacement percentage reduced the permeability of concrete.
[103]	5–30%	CCA enhanced the sulfate resistance of concrete.
[104]	0–25%	CCA was suitable to replace some cement in cement mortars as an insulation material.
[105]	0%, 20%, and 50%	The 20% CCA in concrete improved water absorption and durability of specimens without affecting the strength significantly.
[106]	2%, 4%, and 6%	The use of CCA in cement concrete reduced water absorption and enhanced the resistance to sulfuric acid attack.
[96]	10%, 20%, 30%, and 40%	The water absorption rate was 2.15% when cement was substituted by CCA at the proportion of 40% compared to 3.88% of the control group.

**Table 13:** The chemical composition of SCBA.

Ref.	SiO_2_	Al_2_O_3_	Fe_2_O_3_	CaO	MgO	Na_2_O	K_2_O	SO_3_	LOI (%)
[112]	61.00	4.99	9.20	4.40	2.79	0.15	6.98	3.85	2.08
[39]	74.34	3.40	5.55	2.15	0.67	0.12	1.46	-	11.42
[113]	75.67	2.29	1.52	6.62	1.87	0.12	9.59	-	3.00~6.00
[114]	72.95	1.89	1.68	7.77	1.98	-	9.28	4.45	21.00
[115]	70.00	6.50	13.30	2.10	-	-	4.10	2.40	0.30
[116]	78.34	8.55	3.61	2.15	1.65	0.12	3.46	-	0.42
[117]	55.70	2.86	3.51	15.34	4.08	0.37	6.10	-	8.92

**Table 14:** Physical properties of SCBA.

Ref.	Median Particle Size (μm)	Specific Gravity (g/cm^3^)	Specific Surface Area (m^2^/g)
[114]	-	1.91	0.145
[118]	-	2.2	0.514
[119]	30	2.22	-
[120]	6.63	2.04	0.696
[111]	76.3	-	0.196
	12.0	-	0.444
	2.7	-	0.893
[121]	2.12	-	0.453

**Table 16:** Effect of SCBA on the durability of cement mortar and concrete.

Ref.	Replacement Level	Effect
[130]	0% and 20%	The chloride ion resistance of 20% SCBA mixed concrete was significantly improved compared to concrete.
[113]	0%, 5%, 10%, 15%, 20%, and 25%	The addition of SCBA in concrete enhanced its resistance to chloride ions.
[131]	0%, 10%, 20%, and 30%	The addition of SCBA with low LOI in concrete reduced water absorption.
[132]	10%, 20%, 30%, 40%, and 50%	The charge passed of concrete containing 50% SCBA decreased from 4181 C to 304 C, significantly improving its resistance to chloride ion corrosion.
[133]	10–30%	SCBA enhanced durability of cement mortar by improving its pore structure.
[122]	5–30%	20% SCBA reduced the porosity of the concrete, thereby reducing the water absorption rate.
[134]	10%, 20%, and 30%	The use of 10% SCBA decreased the drying shrinkage of cement mortar by 8% compared to the cement mortar.

**Table 17:** The chemical composition of BLA.

Ref.	SiO_2_	Al_2_O_3_	Fe_2_O_3_	CaO	MgO	Na_2_O	K_2_O	SO_3_	LOI (%)
[142]	78.71	0.54	1.01	7.82	1.83	0.05	3.78	1.00	3.83
[139]	80.40	0.71	1.22	5.06	0.99	0.08	1.33	1.07	8.04
[143]	72.97	2.31	2.85	4.98	1.23	-	6.07	0.55	4.20
[140]	74.70	0.15	0.21	4.48	3.23	0.56	5.14	4.18	3.98
[144]	70.50	0.632	0.468	7.86	1.84	-	5.14	2.87	7.79
[145]	75.90	4.13	1.22	7.47	1.85	0.21	5.62	1.06	-
[23]	65.66	6.41	4.28	15.22	2.48	2.76	4.84	-	9.65

**Table 18:** Effect of BLA on the mechanical properties of cement mortar and concrete.

Ref.	Replacement Level	Mix Type	Effect
[144]	0%, 10%, and 20%	cement mortar	The compressive strength of cement mortar containing 20% BLA increased by 19% and 31% after 7 d and 28 d, respectively.
[149]	0%, 5%, 10%, and 15%	high-performance concrete (HPC)	The compressive strength of HPC with 10% BLA at 56 days was similar to that of the control group.
[147]	0%, 5%, 10%, 15%, 20%, and 25%	high-performance concrete (HPC)	5% BLA enhanced the compressive strength of HPC by more than 30%.
[150]	5%, 10%, and 15%	concrete	The compressive strengths of BLA5, BLA10, and BLA15 were less than that of the control mix by 11%, 21%, and 41%, respectively.
[148]	10%, 20%, and 30%	self-compacting concrete (SCC)	The mechanical properties of BLA partially replacing SCC decreased.
[151]	0%, 10%, and 20%	cement mortar	The compressive strength of 20 wt.% BLA mixed cement mortar was similar to that of ordinary cement mortar at 28 d.
[141]	5–30%	cement mortar	Replacement of OPC by BLA (5–30%) was achieved, yielding good mechanical performance.

**Table 19:** Effect of BLA on the durability of cement mortar and concrete.

Ref.	Replacement Level	Effect
[152]	0%, 10%, 15%, and 20%	The sample with a BLA content of 10% exhibited excellent performance, with higher acid resistance and lower corrosion rate.
[153]	0%, 5%, 10%, and 15%	The addition of BLA in concrete enhanced its resistance to acid attack.
[154]	0%, 20%, and 30%	The use of BLA reduced the mass loss of cement mortars after acid erosion, but the strength slightly decreased compared to the control group.
[23]	5%, 10%, 15%, and 20%	The water absorption and erosion resistance of concrete with 10% BLA had significant improvement.
[149]	0%, 5%, 10%, and 15%	In sulfate solution, concrete with 5% BLA had a strength loss rate of 37.26%, lower than the control (40.01%).

**Table 20:** The chemical composition of EGA.

Ref.	SiO_2_	Al_2_O_3_	Fe_2_O_3_	CaO	MgO	Na_2_O	K_2_O	SO_3_	LOI (%)
[156]	59.60	5.90	22.10	2.60	-	-	3.50	1.80	3.00
[157]	80.00	0.77	0.44	1.85	1.11	-	7.05	0.65	6.31
[158]	55.10	22.10	9.20	2.50	-	-	5.00	1.80	1.10
[160]	49.40	0.47	0.83	10.40	4.22	-	8.60	0.47	14.60
[161]	70.74	0.34	0.29	5.96	10.81	0.07	16.99	4.74	-
[159]	70.20	0.52	0.85	5.30	4.87	0.18	9.06	3.61	1.45

**Table 21:** Physical properties of EGA.

Ref.	Median Particle Size (μm)	Specific Gravity (g/cm^3^)	Specific Surface Area (m^2^/g)
[156]	11.6	2.62	42.1
	10.0	2.60	44.3
	10.8	2.51	72.6
[157]	25.49	-	-
	49.02	-	-
[158]	7.6–13.2	2.48	40.3
		2.49	36.4
		2.55	20.3

**Table 22:** Effect of EGA on the mechanical properties of cement mortar and concrete.

Ref.	Replacement Level	Mix Type	Effect
[156]	35%	concrete	The mechanical properties of concrete prepared with 35% BLA are almost the same as those of the control group.
[160]	20%	concrete	EGA was suitable to be used as a pozzolanic material in cement.
[162]	10%, 15%, and 20%	concrete hollow blocks (CHB)	EGA was suitable for non-load-bearing CHB.
[163]	20%	cement mortar	The mechanical properties of cement mortars containing 20% EGA were the same as those of the control group.
